# Identification of Histone Modifications Reveals a Role of H2b Monoubiquitination in Transcriptional Regulation of *dmrt1* in *Monopterus albus*

**DOI:** 10.7150/ijbs.59347

**Published:** 2021-05-11

**Authors:** Fengling Lai, Yibin Cheng, Juan Zou, Haoyu Wang, Wang Zhu, Xin Wang, Hanhua Cheng, Rongjia Zhou

**Affiliations:** Hubei Key Laboratory of Cell Homeostasis, College of Life Sciences, Wuhan University, Wuhan 430072, China.

**Keywords:** histone modification, mass spectrometry, H2B ubiquitination, sex reversal

## Abstract

Gonadal trans-differentiation from ovary to testis occurs in a same individual, suggesting a role of epigenetic regulation. However, histone modifications concerning the sex reversal process remain elusive. We analyzed histone modifications using liquid chromatography-tandem mass spectrometry (LC-MS/MS). Chromatin immunoprecipitation followed by sequencing (ChIP-seq) technology was used to test chromatin immunoprecipitation of gonads. Western blot analysis was performed to analyze protein expression. Immunofluorescence analysis was conducted to localize proteins in gonadal tissues. Here, we report a developmental atlas of histone modifications in the gonadal differentiation, including acetylation, methylation, and ubiquitination. We provided a detail distribution map of these modification sites including novel histone modifications along histones H2a, H2b, H3, and H4, and revealed their relationship with types of gonadal differentiation. We then determined a testis-enriched histone modification site, H2b monoubiquitination at K120, and its association with spermatogenesis. ChIP-seq demonstrated that the modification was highly enriched in the male sex-determining gene *dmrt1* (doublesex and mab-3 related transcription factor 1), in particular, in its exon regions, suggesting its role in transcriptional regulation of* dmrt1* in testis. Together, these data not only provide a new resource for epigenetic study in gonadal development, but also define an association of histone modifications with gonadal differentiation from ovary to testis.

## Introduction

Nucleosomes, as the basic unit of chromatin, consist of 146 base pairs of DNA wrapped around a core of histone octamer in eukaryotes 1. Histone octamer comprises two H2A-H2B heterodimers and an (H3-H4)_2_ tetramer 2. There are five major histone families, including H1/H5, H2A, H2B, H3, and H4 3, which are among the most highly conserved gene family in eukaryotes. The residues in the tails and the histone-fold domains of histone octamer are subject to various post-translational modifications, for example, acetylation, methylation, ubiquitination, phosphorylation, SUMOylation, ADP-ribosylation, biotinylation, carbonylation, glycosylation, deamination, proline isomerization, and cleavage of the histone tail 4-7. These post-translational modifications play important roles in regulating gene expression, chromatin dynamics, DNA repair, and genome stability 5, 7-11.

Epigenetic regulations are involved in the initiation, stabilization, and maintenance of sexual differentiation in vertebrates. Epigenetic marks, DNA methylation and histone modifications, showed differences between testis and ovary, in mammals 12, chicken 13, fishes 14, 15, turtle, and alligator 16, 17, regardless of genetic or temperature-dependent sex determination, indicating an importance of epigenetic marks for regulation of gonadal differentiation. In mammals, *SRY* (sex-determining region Y gene) is a dominant male sex-determining gene on chromosome Y. An unmethylated CTCF-binding site was mapped to upstream of the human *SRY* gene in white blood cells and was associated with enrichment of histone H3 lysine 9 trimethylation (H3K9me3) marks 18. Consistent with the observation, activation of *Sry* required depletion of H3K9me3 in testis, and XY mice with JMJD1A deficiency, a H3K9-demethylating enzyme, showed a high frequency of sex reversal, with an increase in H3K9 di-methylation and a decrease in H3K4 trimethylation across the locus 19. In mice, loss of histone acetyl-transferases p300 and CBP affected histone acetylation, H3K27ac in particular, at the *Sry* promoter, and caused XY gonadal disorder 20. Histone acetylation facilitated the transition of nucleosomal histones by the phosphorylated protamine 21. Molecular and genetic evidences showed that the epigenetic regulator Kdm6b was essential for male sex determination by demethylating of H3K27me3 at the promoter of *Dmrt1* in turtle 22. In addition, mutations of sex determining gene, Sex lethal (*Sxl*), affected H4 acetylation and gene expression on both the X and autosomes 23. In zebrafish, H3K4me3/H3K27me3 modifications were associated with gene promoter regions in gametes and early embryos, indicating roles of histone modification reprogramming in early embryo development, and H3K4me3 and H3K27me3 modifications were also enriched in the sperm chromatin 24, suggesting important roles of these marks for sperm functions.

Monoubiquitylation of histone H2B on lysine 120 in mammals (lysine 123 in yeast) regulated various molecular processes, for example, DNA damage response and homologous recombination 25-28, transcription initiation and elongation 29-32, DNA replication 33, nucleosome positioning 34, and RNA processing and export 35-39. Histone H2B monoubiquitination at K120 also regulated meiotic recombination 40, preimplantation development 41, embryonic stem cell differentiation 42, as well as regulation of autophagy 43. H2B monoubiquitination can affect chromatin structure and gene transcription, together with H3K4me3 and H3K79 methylation modifications 44. In addition, monoubiquitylation of histone H2B and methylation of histone H3K36me2/3 and H3K79me2/3 co-regulated gene expression 45. These studies indicated that multiple histone modifications along with H2B monoubiquitination were involved in regulating a common or distinct biological process.

In this study, we presented a developmental landscape of histone modifications in gonadal differentiation from ovary to testis, via the ovotestis in *Monopterus albus*, a model fish species for development, genetics and evolution 46. It belongs to the family Synbranchidae of the order Synbranchiformes (Neoteleostei, Teleostei, and Vertebrata) and distributes mainly in southern and eastern Asia, northern Australia, and southeastern United States 47. Recently, our group have sequenced the whole genome and determined its genome size of ~800 Mb 48. Its natural sex reversal characteristic, from female via intersex into male during its life cycle, is an ideal model for studies of sexual differentiation. However, epigenetic regulation concerning the sex reversal process remains unknown. We determined the sequences of histone genes* h1, h2a, h2b, h3,* and* h4,* as well as histone variants *h2a2.2, h2a.v, h2ax, h2a.z,* and *h3.3*, by sequencing and phylogenetic analysis. Using liquid chromatography-tandem mass spectrometry (LC-MS/MS) approaches, we provided a full atlas of post-translational modifications on gonadal histones, including acetylation, methylation, and ubiquitination, along with novel histone modification sites, during gonadal differentiation. We then focused on testis-enriched histone modification site, H2b monoubiquitination at K120, and found that this monoubiquitination modification was associated with spermatogenesis via regulation of transcription of sex-determining gene *dmrt1* in testis.

## Materials and Methods

### Animals

Swamp eels (*Monopterus albus*) were obtained from the Wuhan area in the Yangtze River basin. Their sexes were confirmed by microscopic analysis of gonad sections. All animal experiments and methods were performed in accordance with the relevant approved guidelines and regulations, as well as under the approval of the Ethics Committee of Wuhan University.

### Antibodies and reagents

The primary antibodies were as follows: Rabbit antibody to Ubiquityl-Histone H2b (Lys 120) (Cat# 5546s) was purchased from Cell Signaling Technology, Danvers, MA, USA. Anti-H2b (Cat# ET1612-25) was purchased from HuaAn Biotechnology, Hangzhou, China. Anti-H3 (Cat# 17168-1-AP) was from Proteintech Group, Rosemont, IL, USA. Monoclonal anti-Vasa (Cat # 128306) was purchased from Gene Tex, San Antonio, TX, USA.

The following secondary antibodies were used: horseradish peroxidase (HRP) conjugated-goat anti-Rabbit IgG (H + L) secondary antibody (Cat# 31460) was from Pierce Biotechnology Company, Rockford, IL, USA. The fluorescent TRITC-conjugated ImmunoPure goat anti-Rabbit IgG (H+L) (Cat# ZF-0316) and FITC-conjugated ImmunoPure goat anti-rabbit IgG(H+L) (Cat# ZF-0311) were purchased from Feiyi Technology, Wuhan, Hubei, China. Hoechst (Cat# C1022) was purchased from Beyotime Institute of Biotechnology, Nantong, Jiangsu, China. DAPI (Cat# 1155MG010) was purchased from Biofroxx, Germany.

### Identification of histones and histone variants

Most sequences of the histones and histone variants were obtained from NCBI (https://www.ncbi.nlm.nih.gov/) (accession numbers: histones in Table S1; histone variants in Table S2). The sequence of histone* h3* was amplified by RT-PCR and cloned into the pGEM-T easy in this study (accession number in Table S1). Phylogenetic analysis was used to determine the sequence clusters. We searched homologs of the histones in other species including human, mouse, zebrafish (*Danio rerio*), tilapia (*Orepchromis niloticus*), spotted gar (*Lepisosteus oculatus*), and fugu (*Takifugu rubripes*) in NCBI or Ensembl. We aligned all histones using the full-length proteins by ClustalW, and constructed phylogenetic tree using the maximum-likelihood method in 100 bootstrap replicates by Mega 5. All protein IDs and NCBI accession numbers were listed in Table S1 and S2.

### RT-PCR analysis

Total RNA samples were isolated from adult gonads using TRIzol reagent (Invitrogen, CarIsbad, CA, USA) according to the manufacturer's instructions. Using the MMLV system (Promega, Madison, WI, USA), cDNAs were reversely transcribed from the RNAs. PCR was used to amplify sequences of histone genes,* dmrt1* and *hprt* using the cDNAs as templates. PCR conditions and primer sequences were listed in Supplementary Table S3.

### Histone isolation

Histones were extracted using the acid extraction method 49. In brief, 25 mg of gonadal sample were homogenized in 1 mL hypotonic lysis buffer (Tris HCl 10 mM, pH 8.0; KCl 10 mM; MgCl_2_ 1.5 mM; DTT 1 mM). After centrifuged at 2000 g for 10 min at 4 °C, and the samples were lysed in the hypotonic lysis buffer with 10 % NP-40. Histones were extracted with 0.2 M H_2_SO_4_, precipitated with 100 % TCA and washed with ice-cold acetone. The histone samples were separated with 12 % SDS-polyacrylamide gel and then stained with Coomassie brilliant blue R250.

### Western blot analysis

Western blot analysis was performed as previously described 50. Briefly, protein extracts from ovary, ovotestis, and testis were separated with 12 % SDS-PAGE and transferred onto PVDF membranes (Millipore, Bedford, MA, USA). After blocked with 5 % non-fat milk in TBST for 30 min, the membranes incubated with the primary antibodies (1:1000 dilution in 5 % BSA) for overnight at 4 °C, washed with TBST (20 mM Tris-HCl pH 7.5, 150 mM NaCl, 0.1 % Tween 20) for 5 times, then incubated with HRP-labeled secondary antibody (1:5000 dilution in 5 % non-fat milk) for 1 h at room temperature. After washed with TBST for 5 times, the signals were detected with the ECL kit (Thermo Scientific, Waltham, MA, USA).

### Immunofluorescence analysis

Immunofluorescence analysis was performed as previously described 51. Briefly, fresh gonadal samples were cut into 7 µm sections using a microtome-cryostat (CM1850, Leica, Germany). Tissues sections were fixed with 4 % cold paraformaldehyde (PFA) for 30 min and washed with PBS (pH 7.4) for 3 times. The sections were treated with 1 % Triton X-100 for 25 min and rinsed in PBS for 3 times. After blocking with 5 % bovine serum albumin (BSA) for 20 min, the sections were incubated with primary antibody at 1:100 in 5 % BSA at 4 °C for overnight. After washing in PBS, the sections were incubated with a fluorescein isothiocyanate conjugated secondary antibody at dilution of 1:100 for 1 h at 37 °C, and washed in PBS for 3 times away from light. The nuclei were stained with Hoechst (Cat# C1022) for 5 min and rinsed 3 times for 5 min in PBS. Images were collected immediately using confocal microscope (SP8, Leica).

### Mass spectrometry

To identify *in vivo* histone modification sites, histones were acid extracted from gonadal tissues. After SDS-PAGE and stained with Coomassie brilliant blue R250, the gels with histones H2a, H2b, H3, and H4 were cutting into small pieces, respectively. The histones were subjected to in-gel trypsin complete digestion at a final concentration of 10 ng/μl for overnight at 37 °C and desalted with ZipTip C18 tips (Millipore). The samples were dried with a SpeedVac and dissolved in 50 % ACN/0.1 % TFA. Then, the peptides of histones were analyzed by liquid chromatography-tandem mass spectrometry (LC-MS/MS) on a Q Exactive-HF mass spectrometer (Thermo Fisher Scientific, Waltham, Massachusetts, USA). The LC-MS/MS data were processed using Proteome Discoverer (Thermo Fisher Scientific, Waltham, Massachusetts, USA) via searching against the protein sequence database of *Monopterus albus* (https://www.ncbi.nlm.nih.gov/).

### ChIP sequencing and analysis

Gonadal samples were cross-linked by 1 % formaldehyde. After lysed with lysis buffer (50 mM Tris-HCl pH 8.0, 5 mM EDTA, 0.1 % SDS) and washed with digestion buffer (50 mM Tris-HCl, pH 7.6, 1 mM CaCl_2_, 0.2 % Triton X-100), whole cell lysates were digested by microsphere nuclease of 2×10^3^ gel unit/ml (New England Biolabs (Beijing), Beijing, China). The genomic DNA was sonicated into 100-500 bp. The DNA samples of 20 μg were incubated with beads conjugated with primary antibody or IgG beads at 4 °C for overnight. After washed, the beads were used to extract genomic DNA with Kit from TIANGEN (Beijing, China). After ligation of methylated sequencing adaptor and PCR amplification, 100-300 bp size of DNA sequences were used for ChIP-seq DNA library construction, and sequenced on Illumina HiSeq2000 (Illumina, San Diego, California, USA). After filtering to remove low quality of reads, clean sequences were mapped to reference genome (https://www.ncbi.nlm.nih.gov/). Alignment results were used to call peak, and the peak information was used for comparison analysis between testis and ovary samples.

## Results

### Identification of post-translational modifications on gonadal histones by LC-MS/MS

**Figure 1:**
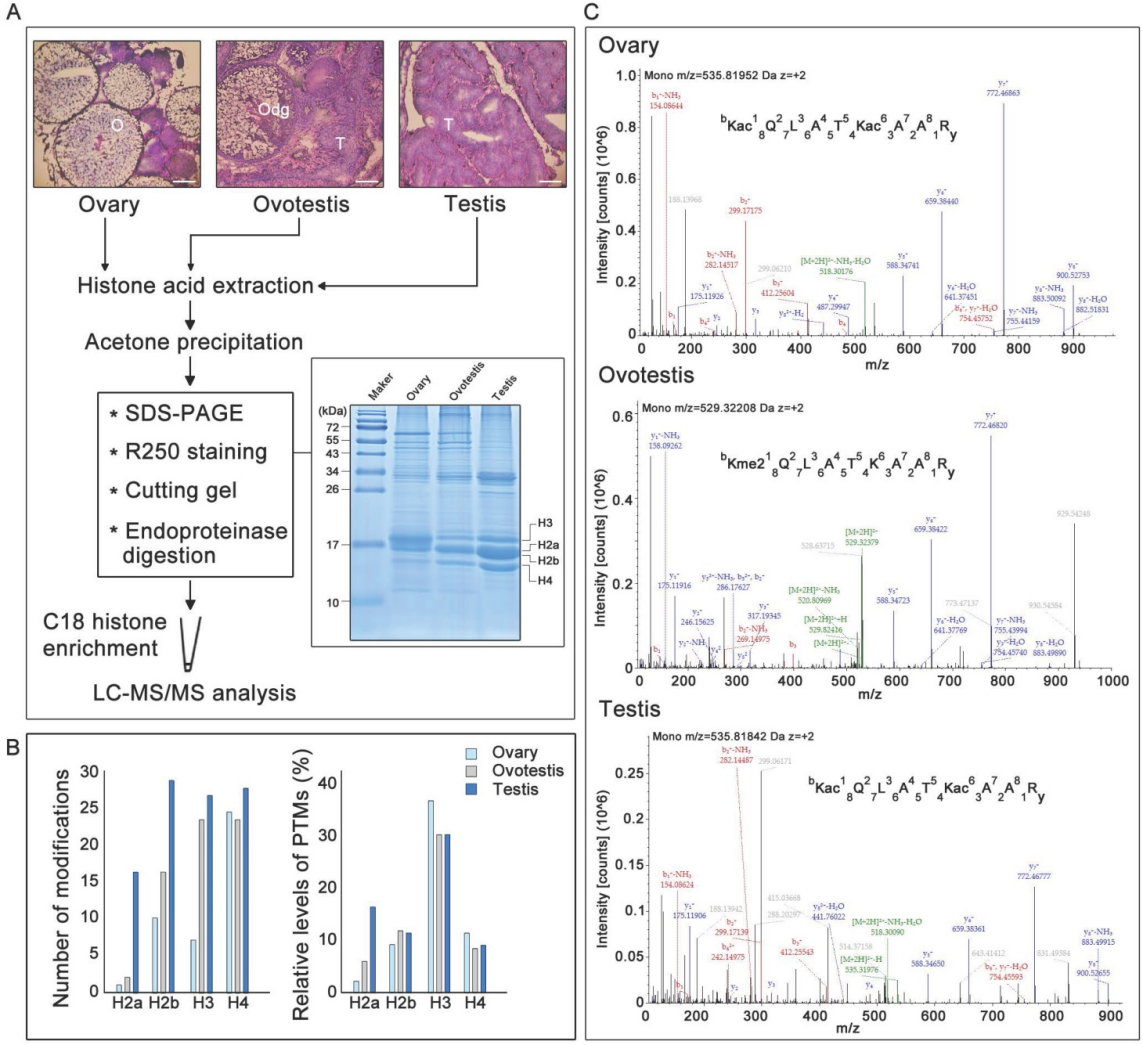
** Gonadal histology and identification of histone modifications by LC-MS/MS.** (A) Histological analysis of ovary, ovotestis, and testis by H&E. staining. O, ovary; Odg, degrading follicles; T, testis. Scale bar, 50 µm. Histones were extracted using acid extraction, resolved by 12 % Tricine-SDS-PAGE, and stained with Coomassie blue. The bands containing histones were cut into pieces based on size and digested by trypsin. The peptide segments were recovered and enriched by C18 column, and the peptide modifications were analyzed by LC-MS/MS. (B) Left panel indicates total number of H2a, H2b, H3, and H4 modification sites, including acetylation, methylation, and ubiquitination, among ovary, ovotestis, and testis. Right panel shows relative levels of modified sites normalized by total peptides with the sites, including acetylation, methylation, and ubiquitination, among ovary, ovotestis, and testis. (C) Mass spectrometry of H3K18 acetylation or dimethylation in the peptide covering residues 18-26 of histone H3 (KQLATKAAR) isolated from gonads. Top panel, annotated MS/MS spectrum of acetylation at H3K18 and K23 in ovary. Middle, annotated MS/MS spectrum of dimethylation at H3K18 in ovotestis. Bottom, annotated MS/MS spectrum of acetylation at H3K18 and K23 in testis. b: N-terminal fragment ion series; y: C-terminal fragment ion series.

Before identification of histone modifications, genes encoding histones and their variants were first characterized in the swamp eel genome. Phylogenetic trees for histone and variant genes in vertebrates were constructed (Supplementary Figure S1, Supplementary Tables S1 and S2). Five histone variant genes, *h2a2.2, h2a.v, h2ax, h2a.z,* and *h3.3*, in addition to five histone genes *h1, h2a, h2b, h3,* and* h4,* were identified in the species, based on sequence identity and clustering (Supplementary Figures S1 and S2; Supplementary Tables S1 and S2). To identify potential histone modifications during gonad differentiation from ovary to testis via an ovotestis transition (Figure 1A), gonadal histones were then identified. SDS-PAGE showed that histones had various abundance among ovary, ovotestis and testis, and among histones within a gonad (Figure 1A). Liquid chromatography-tandem mass spectrometry of histones after trypsin digestion was used to further identify potential histone modification sites. As all peptide cleavage by trypsin occurs only on lysine and arginine residues, different sizes of digested histone peptides with lysine or arginine at the C-terminal could be obtained for LC-MS/MS. Overall number of modification sites on H2a, H2b, H3, and H4, including acetylation, methylation, and ubiquitination, indicated that an increasing trend of modifications during gonad differentiation from ovary to testis via ovotestis (Figure 1B). However, percentage of modified sites showed that the highest relative level (percentage) of modified histone sties, in ovary in particular, were detected on H3, and only H2a had an increasing trend from ovary to testis, while no obvious difference was observed among gonads on H2b and H4 (Figure 1B). In addition, some amino acid residues on the same peptide were subject to different types of modifications among ovary, ovotestis and testis, for example, H3K18 was acetylated in ovary, ovotestis, and testis, but dimethylated in ovotestis (Figure 1C), suggesting differential histone modifications during gonad differentiation.

### Testis-biased histone modifications

**Figure 2:**
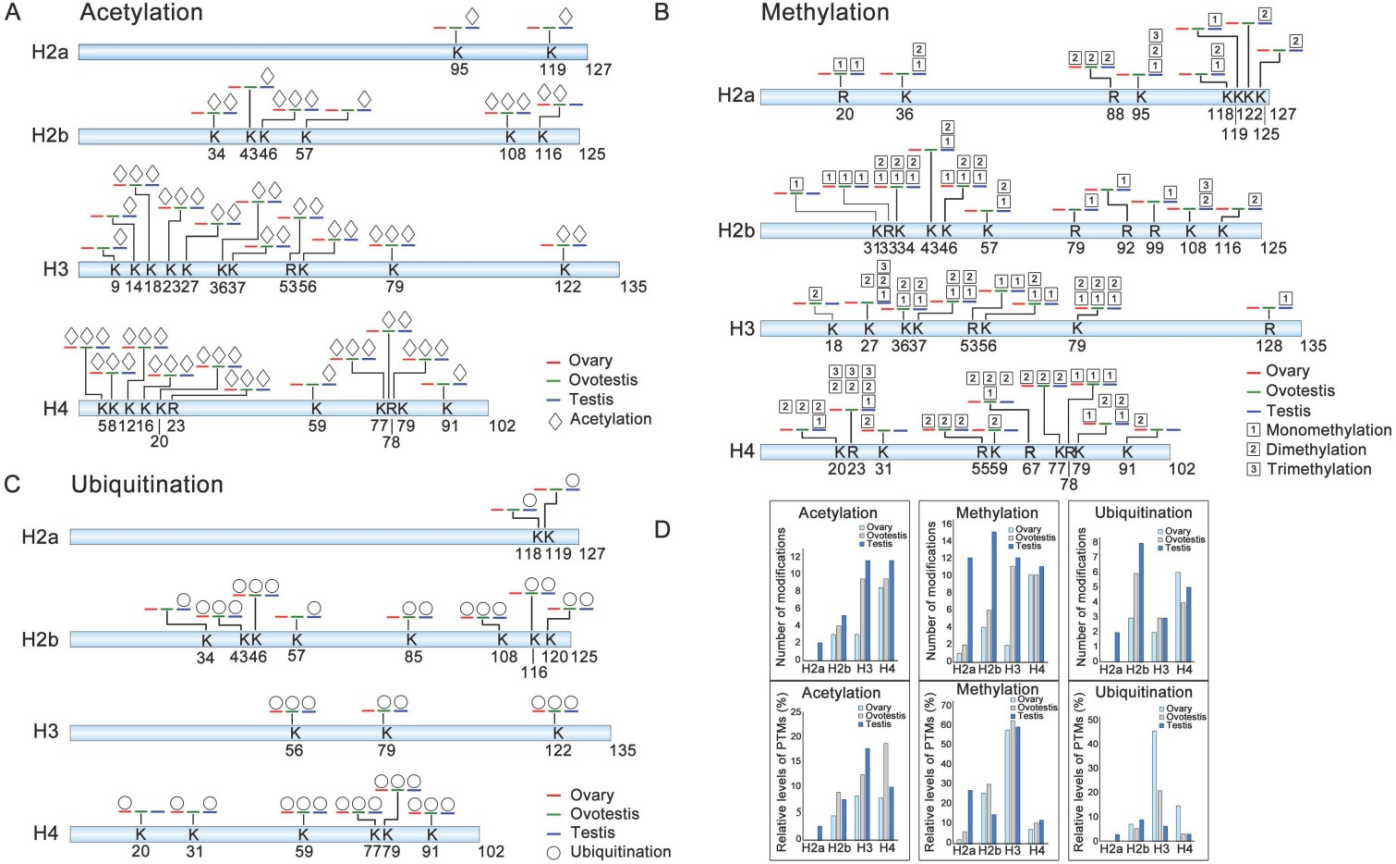
** Identification of post-translational modification sites on histones among ovary, ovotestis, and testis.** (A) Distribution of histone acetylation sites on histones H2a, H2b, H3, and H4 in gonads. (B) Methylation sites identified on H2a, H2b, H3, and H4 in gonads. (C) Ubiquitination sites on H2a, H2b, H3, and H4 in gonads. Symbol key: the number represents the amino acid site of the peptide; the K is for lysine while the R for arginine; the blue line represents “testis”; the green line indicates “ovotestis”; the red line denotes “ovary”; rhombus is for acetylation; squares with 1, 2 and 3 are for mono-, di- and tri-methylation, respectively; circle is for ubiquitination; (D) Upper panel indicates the number of acetylation, methylation and, ubiquitination modification sites on H2a, H2b, H3, and H4 among ovary, ovotestis, and testis, respectively. Lower panel shows relative levels of modified sites normalized by total peptides with the sites, including acetylation, methylation, and ubiquitination, among ovary, ovotestis, and testis, respectively.

To explore differential histone modifications among ovary, ovotestis and testis, LC-MS/MS were used to identify all sites of acetylation, methylation, and ubiquitination on H2a, H2b, H3, and H4. Histone acetylation sites were mainly distributed on H3 and H4, but less on H2a and H2b. There were 8 testis specific acetylation sites, and another 8 sites can be detected in both ovotestis and testis, for example H3K9ac and H3K27ac (Figure 2A). Moreover, for acetylation modification, an increasing trend towards testis was obvious on H3, and the highest relative level of acetylation occurred on H4 in ovotestis (Figure 2A,D). This suggested that acetylation modifications were involved in testis differentiation. Indeed, transcriptional activation was associated with the modifications of H3K9ac 52 and H3K27ac 53.

We identified three histone methylation states, mono-methylation, dimethylation, and trimethylation. Of 37 histone methylated sites, 14 sites were testis specific, and 6 sites were detected in both ovotestis and testis (e.g. H3K27 and H3K36), 3 sites (H2bK31, H3K18, and H4K59) were ovotestis specific, and 2 sites (H4K31 and H4K91) were ovary specific (Figure 2B). Methylation modification was abundant on H3 among gonads, and was high on H2a in testis in comparison with both ovotestis and ovary (Figure 2B,D). A small number of methylation modification sites were related to transcriptional silencing, for example, H3K27, but many modification sites were associated with transcriptional activation, such as H3K4, H3K36, and H3K79 10, 22. Thus, these results suggested that methylation modification was associated with gonad differentiation.

For ubiquitination modification, 19 sites were identified. Of which, 4 sites were testis specific (e.g. H2aK118 and H2aK119), 4 sites were in both ovotestis and testis (e.g. H2bK120) (Figure 2C). Moreover, the highest relative level of ubiquitination sites was observed on H3 in ovary, although number of ubiquitination sites detected was high on H2b, in testis in particular (Figure 2D). Some of these modified sites were associated transcriptional regulation, for example, H2bK120 and H2aK119 36, 54.

### Distribution feature of modification sites in histone fold domain and tail regions

**Figure 3:**
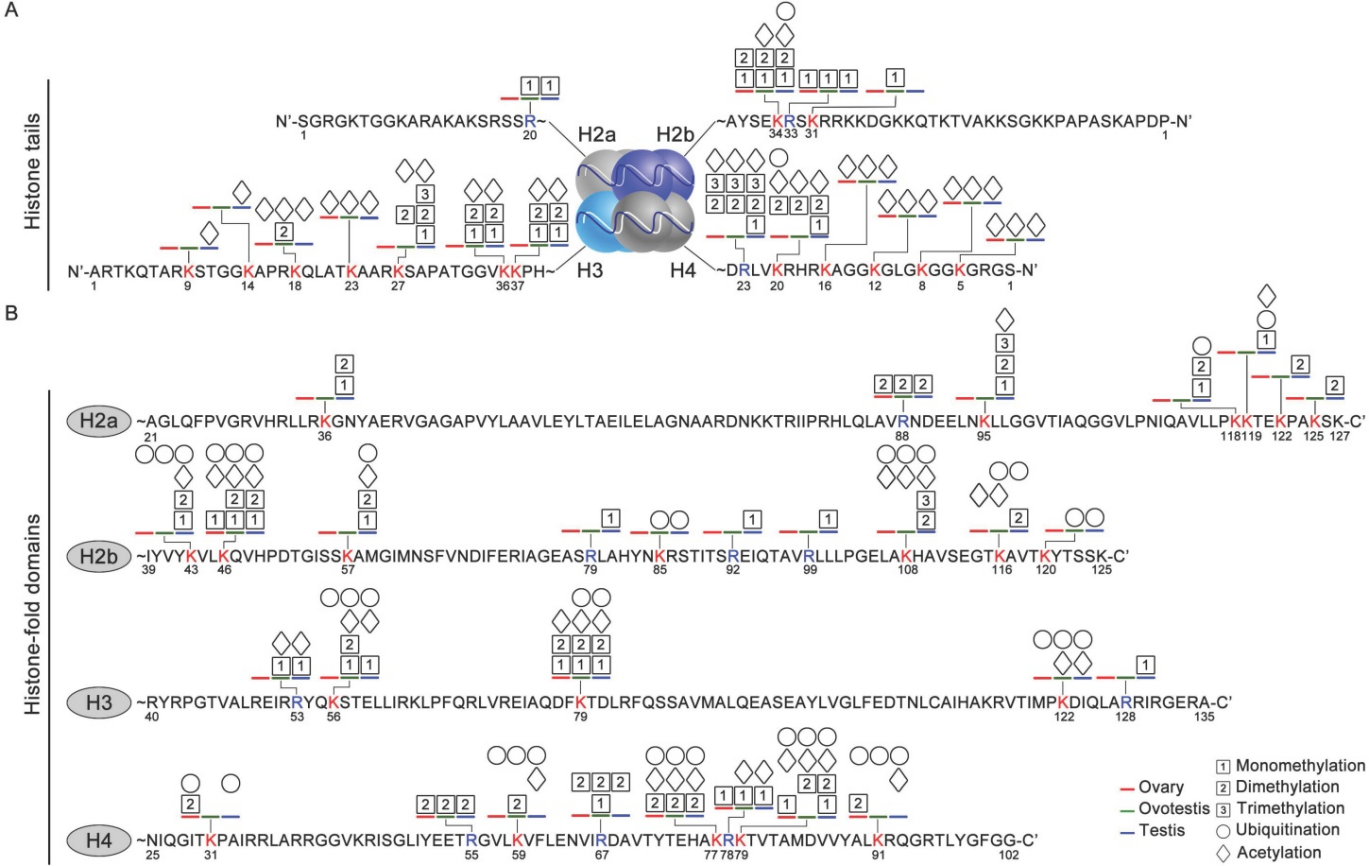
** Various modification sites at histone tails and in histone-fold domains among gonadal tissues.** (A) Distribution of modifications at the N-ter histone tails, extended from the globular core of histones H2a, H2b, H3, and H4. DNA is wrapped around the nucleosome octamer made up of two H2a-H2b dimers and a H3-H4 tetramer. (B) Distribution of modification sites in histone-fold domains of H2a, H2b, H3, and H4. Post-translational modifications: acetylation (rhombus), methylation (squares with 1, 2, and 3 represent mono-, di- and trimethylation, respectively) and ubiquitination (circle). The numbers represent the amino acid sites in the peptide; the blue line, testis; the green line, ovotestis; the red line ovary; Blue R, arginine residues; Red K, lysine residues.

As histone code at the tail was associated with epigenetic regulations of various cellular activities 55, we analyzed distribution of histone modification sites in both histone tail and fold domain. At the tails, a higher density of modification sites was detected on both H3 and H4, compared to the fold domains (Figure 3A,B). Moreover, modification sites occurred more frequently at the tails of both H3 and H4, than those of both H2a and H2b. In addition, most of these modifications were acetylation and methylation at the tails of H2a, H2b, H3 and H4 (Figure 3A). Ubiquitination modifications were mainly detected in the fold domains, and only 2 sites were observed at the tails of the histones (Figure 3B). These data suggested that these distinct modifications were probably involved in gonad differentiation.

### Histone H2b monoubiquitination at K120 is associated with testis differentiation

**Figure 4:**
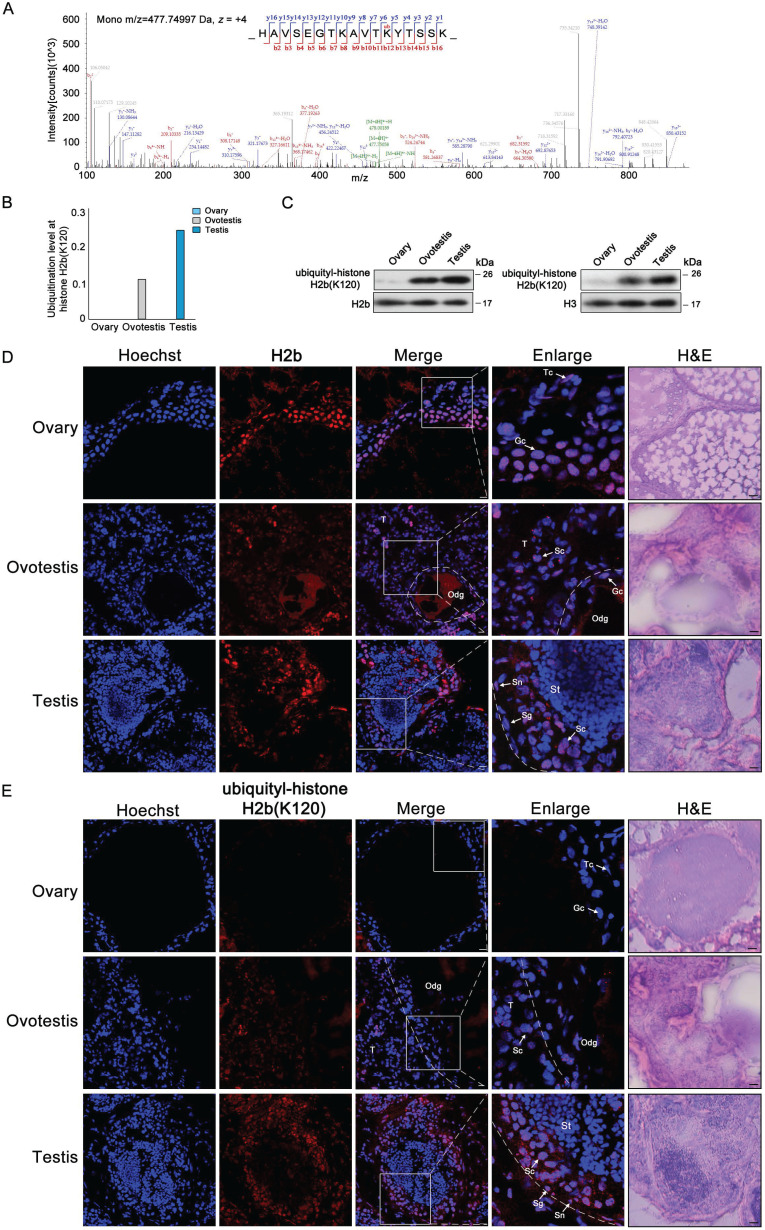
** Ubiquitylated H2b at K120 associated with spermatogenesis.** (A) Mass spectrum of H2b peptide showing H2bK120 ubiquitylation. m/z, mass/charge ratio. (B) Ubiquitylation levels at H2b (K120) in ovary, ovotestis, and testis. The ratio of modified peptides to unmodified peptides are indicated. (C) Western blot analysis of the histone ubiquitylation level using the ubiquitylation-histone H2b (K120) antibody. Total protein samples were isolated from ovary, ovotestis, and testis. H2b or H3 were used as an internal control, respectively. (D) Immunofluorescent analysis of H2b in ovary, ovotestis and testis using anti-H2b antibody. (E) Immunofluorescent analysis of ubiquitylated histone H2b at K120 in ovary, ovotestis and testis using anti-ubiquitylated histone H2b at K120 antibody. The nuclei were stained by Hoechst (blue). The enlarged image originated from the region with white square. Sn, Sertoli cells; Sg, spermatogonia; Sc, spermatocytes; Tc, theca cells; Gc, granulosa cells; St, spermatids. Odg, degrading follicles; T, testis. Scale bar, 5 µm.

As H2b monoubiquitination K120 (H2bK120ub) was detected in both testis and ovotestis (Figures 2C and 4A), we further investigated whether H2bK120ub is involved in testis differentiation. H2b monoubiquitination occurred at K120 site in the peptide covering residues 109-125 (HAVSEGTKAVTKYTSSK) of histone H2b (Figure 4A). Based on mass spectrometry data, detected peptides with H2b monoubiquitination were mostly enriched in testis, moderately in ovotestis, but scarcely in ovary (Figure 4B). Western blot analysis using anti-H2bK120ub specific antibody confirmed the observation (Figure 4C), suggesting that H2b monoubiquitination at K120 was abundant in testis. To further determine cell types with H2bK120ub modification in testis, immunofluorescent analysis of ubiquitylated histone H2b at K120 using anti-H2bK120ub antibody showed that the signals of TRITC-labeled H2bK120ub were observed in Sertoli cells, spermatogonia, and spermatocytes, but not in the spermatids in testis, while no signal was detected in ovary (Figure 4D,E). Immunofluorescence using anti-Vasa (a marker of germ cells) confirmed the expression pattern of H2bK120ub in testis (Supplementary Figure S3). These results suggested that H2bK120ub was associated with spermatogenesis.

### Histone H2b monoubiquitination at K120 associated with transcription of sex-determining gene *dmrt1* in testis

**Figure 5:**
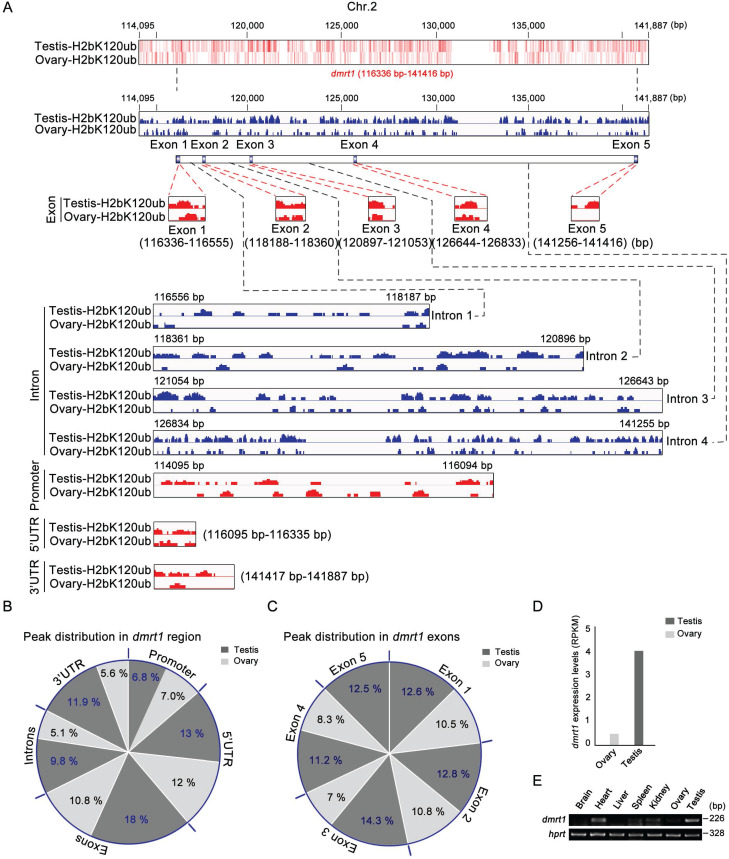
** Ubiquitination of histone H2b (K120) in the chromatin region of *dmrt1* gene related to its expression.** (A) Heatmap and bar chart of ubiquitination levels of histone H2b (K120) in the chromatin region of *dmrt1* gene in ovary and testis. The number represents the base position of *dmrt1* on chromosome 2. Ubiquitination levels are indicated in exons, introns, promoter, 5' UTR, and 3' UTR regions of *dmrt1* between testis and ovary. (B) Percentage of ubiquitination of histone H2b (K120) among exons, introns, promoter, 5' UTR, and 3' UTR regions of *dmrt1* between testis and ovary. The values indicate the ratio of reads to the base length in the region. (C) Percentage of ubiquitination of histone H2b (K120) among exons of *dmrt1* between testis and ovary. The dark gray, testis; the light gray, ovary. (D) Relative expression of *dmrt1* in testis and ovary by RNA-seq. RPKM, reads per kilobase per million. RNA-seq datasets were downloaded from NCBI Gene Expression Omnibus (GEO; http://www.ncbi.nlm.nih.gov/geo/) under accession number GSE43649 48. (E) RT-PCR analysis of *dmrt1* expression in adult tissues. *hprt* was used as an internal control.

As sex-determining gene *dmrt1* was expressed in Sertoli cells and spermatogonia, and required for spermatogenesis in mice 56, and H2bK120ub modification was enriched in these cell types, we explored potential association of H2bK120ub modification with *dmrt1* expression in gonads. ChIP-seq technology was used to explore chromatin regions enriched with H2bK120ub in both testis and ovary with H2bK120ub antibody to obtain the genes related to H2bK120ub modification. We observed that number of ChIP-seq reads in the *dmrt1* gene region on chromosome 2 was obviously high in testis than ovary (Figure 5A). Different regions of *dmrt1* had distinct peak distribution, with higher enrichment in the exons and 3' UTR region in testis than ovary, although less peaks in promoter, the 5 'UTR region, and introns in both testis and ovary (Figure 5B,C). High enrichment of H2bK120ub modification in the* dmrt1* region suggested that chromatin relaxing was involved in transcription regulation of* dmrt1* in testis, not ovary. Thus, we further confirmed that *dmrt1* was highly expressed in testis in comparison with ovary by RT-PCR and RNA-seq data (Figure 5D,E). These results suggested that monoubiquitination of histone H2b at K120 was associated with transcriptional regulation of the sex-determining gene *dmrt1* in testis.

## Discussion

Histone codes, such as methylation, acetylation, ubiquitination, and phosphorylation, etc., that individually or collectively affect gene expression, via activation and repression. Epigenetic regulations, including histone modifications, of sex reversal process in the model fish *Monopterus albus* remains unknown. In this study, we used LC-MS/MS technologies to identify post-translational histone modifications during gonadal differentiation from ovary to testis via the ovotestis and presented a developmental atlas of histone modifications in the gonadal differentiation, including acetylation, methylation, and ubiquitination. Further focusing on histone H2b monoubiquitination at K120, we determined cellular distribution of the ubiquitination modification in testis and its association with spermatogenesis. ChIP-seq demonstrated that the modification was highly enriched in the male sex-determining gene *dmrt1,* especially in exon regions, suggesting its role in transcriptional regulation of* dmrt1* in testis. Together, these results define an association of histone modifications with gonadal differentiation from ovary to testis.

Gonadal trans-differentiation from ovary to testis occurs in a same individual, indicating epigenetic roles, but not genetic change during the developmental process. Our large scale of identifications of post-translational modifications on gonadal histones demonstrated that histone acetylation, methylation, and ubiquitination occurred dynamically during the gonadal differentiation, suggesting important roles of histone modifications in gonadal reconstruction through regulation of transcriptional activation and/or repression. Indeed, we have identified a dose of histone marks for transcriptional activation, including H3K9ac, H3K14ac, H3K18ac, H3K23ac, H3K27ac, H3K36me, H3K79me, H4K20me, H2bK120ub, and H4K31ub, in addition to the marks for transcriptional repression, for example, H3K27me3, H2aK118ub, and H2aK119ub. Acetylation modifications were associated with transcriptional activation, for example, H3K9ac 52, H3K18ac 57, and H3K27ac 53, while methylation and ubiquitination were also involved in gene expression, such as H3K79me 58, H4K20me 59, and H4K31ub 60. In contrast, methylation was mainly related to transcriptional repression, including H3K27me3 61, while some ubiquitination sites were also involved in gene silencing, for example, H2aK118ub and H2aK119ub 62.

A subset of novel marks of histone modifications were also identified in the gonads. In general, the majority of the novel acetylation, ubiquitination, and methylation were enriched in testis, for example, acetylated H2aK95, ubiquitinated H2bK85, and methylated H2aK95. In addition, ovary/ovotestis-biased sites were also detected in ovary (methylated H4K91), ovotestis (methylated H2bK31), and both ovary and ovotestis (acetylated H2bK116). The marks of histone modifications with a strong preference for testis suggested important roles of histone modifications in differentiation from ovary to testis through regulation of gene expression. Ovary-enriched methylation marks could be important for gene silencing during gonadal differentiation through shutdown of certain undesired gene expressions, for example, genes for oogenesis. In addition, H4 trimethylation at arginine 23 was detected in all three kinds of gonads. H4 trimethylation occurs often at lysine, but scarcely at arginine 23. Together, all these histone modifications identified in the study provide a new resource for epigenetic exploration in sexual differentiation. However, further study is needed to elucidate whether and how histone modifications exert their roles in gonadal differentiation.

Identification of the male sex-determining gene *dmrt1* whose expression is associated with H2b monoubiquitination offers a mechanistic explanation of *dmrt1* expression. How *dmrt1* is regulated at transcriptional level remains elusive. In vertebrates, including mammals, chicken, and fishes, *dmrt1* was required for testis differentiation and upregulation of* dmrt1* expression was necessary for male sex differentiation 56, 63, 64. In turtle, histone demethylase KDM6B eliminated trimethylation of H3K27 in the *dmrt1* promoter region, thus enhanced *dmrt1* transcription 22. Transcription factors Sp1 and Egr1 can bind to the *dmrt1* promoter and regulated its transcription in rat Sertoli cells 65. In this study, we determined that H2b monoubiquitination at lysine 120 was upregulated during gonadal differentiation from ovary to testis. ChIP-seq analysis revealed a close association of this modification with transcriptional regulation of *dmrt1* in testis, thus assuring upregulation of* dmrt1* expression in testis differentiation. In fact, histone H2b monoubiquitination has been implicated in global transcriptional elongation in mammalian cells 66 and yeast 67, which were mediated by regulation of nucleosome dynamics during transcription elongation 68. Together, these data suggested that histone H2b monoubiquitination in the region of the *dmrt1* gene facilitates sexual dimorphic expression of *dmrt1* between sexes and afterwards testis differentiation.

## Conclusions

LC-MS/MS reveal post-translational histone modifications during gonadal differentiation from ovary to testis via the ovotestis. A developmental atlas of histone modifications in the gonadal differentiation, including acetylation, methylation, and ubiquitination, is presented. Histone H2b monoubiquitination at K120 is highly enriched in the male sex-determining gene *dmrt1,* especially in exon regions, suggesting its role in transcriptional regulation of* dmrt1* in testis.

## Supplementary Material

Supplementary figures and tables.

## Abbreviations

LC-MS/MS: liquid chromatography-tandem mass spectrometry
ChIP: Chromatin Immunoprecipitation
*dmrt1*: doublesex and mab-3 related transcription factor 1
RT-PCR: reverse transcription-polymerase chain reaction
H2b: histone H2b
H2bub1: H2b monoubiquitination K120

## References

[1] Kornberg RD, Thomas JO (1974). Chromatin structure; oligomers of the histones. Science.

[2] Luger K, Mader AW, Richmond RK, Sargent DF, Richmond TJ (1997). Crystal structure of the nucleosome core particle at 2.8 A resolution. Nature.

[3] Bhasin M, Reinherz EL, Reche PA (2006). Recognition and classification of histones using support vector machine. J Comput Biol.

[4] Ito T (2007). Role of histone modification in chromatin dynamics. J Biochem.

[5] Kouzarides T (2007). Chromatin modifications and their function. Cell.

[6] Shahbazian MD, Grunstein M (2007). Functions of site-specific histone acetylation and deacetylation. Annu Rev Biochem.

[7] Weake VM, Workman JL (2008). Histone ubiquitination: triggering gene activity. Mol Cell.

[8] Berger SL (2007). The complex language of chromatin regulation during transcription. Nature.

[9] Campos EI, Reinberg D (2009). Histones: annotating chromatin. Annu Rev Genet.

[10] Zhou VW, Goren A, Bernstein BE (2011). Charting histone modifications and the functional organization of mammalian genomes. Nat Rev Genet.

[11] Patel DJ, Wang Z (2013). Readout of epigenetic modifications. Annu Rev Biochem.

[12] Miyawaki S, Tachibana M (2019). Role of epigenetic regulation in mammalian sex determination. Curr Top Dev Biol.

[13] Bisoni L, Batlle-Morera L, Bird AP, Suzuki M, McQueen HA (2005). Female-specific hyperacetylation of histone H4 in the chicken Z chromosome. Chromosome Res.

[14] Navarro-Martin L, Vinas J, Ribas L, Diaz N, Gutierrez A, Di Croce L (2011). DNA methylation of the gonadal aromatase (cyp19a) promoter is involved in temperature-dependent sex ratio shifts in the European sea bass. PLoS Genet.

[15] Shao C, Li Q, Chen S, Zhang P, Lian J, Hu Q (2014). Epigenetic modification and inheritance in sexual reversal of fish. Genome Res.

[16] Radhakrishnan S, Literman R, Mizoguchi B, Valenzuela N (2017). MeDIP-seq and nCpG analyses illuminate sexually dimorphic methylation of gonadal development genes with high historic methylation in turtle hatchlings with temperature-dependent sex determination. Epigenetics Chromatin.

[17] Parrott BB, Kohno S, Cloy-McCoy JA, Guillette LJ Jr (2014). Differential incubation temperatures result in dimorphic DNA methylation patterning of the SOX9 and aromatase promoters in gonads of alligator (Alligator mississippiensis) embryos. Biol Reprod.

[18] Singh NP, Madabhushi SR, Srivastava S, Senthilkumar R, Neeraja C, Khosla S (2011). Epigenetic profile of the euchromatic region of human Y chromosome. Nucleic Acids Res.

[19] Kuroki S, Matoba S, Akiyoshi M, Matsumura Y, Miyachi H, Mise N (2013). Epigenetic regulation of mouse sex determination by the histone demethylase Jmjd1a. Science.

[20] Carré GA, Siggers P, Xipolita M, Brindle P, Lutz B, Wells S (2018). Loss of p300 and CBP disrupts histone acetylation at the mouse Sry promoter and causes XY gonadal sex reversal. Hum Mol Genet.

[21] Kurtz K, Saperas N, Ausio J, Chiva M (2009). Spermiogenic nuclear protein transitions and chromatin condensation. Proposal for an ancestral model of nuclear spermiogenesis. J Exp Zool B Mol Dev Evol.

[22] Ge C, Ye J, Weber C, Sun W, Zhang H, Zhou Y (2018). The histone demethylase KDM6B regulates temperature-dependent sex determination in a turtle species. Science.

[23] Bhadra U, Pal-Bhadra M, Birchler JA (2000). Histone acetylation and gene expression analysis of sex lethal mutants in Drosophila. Genetics.

[24] Zhu W, Xu X, Wang X, Liu J (2019). Reprogramming histone modification patterns to coordinate gene expression in early zebrafish embryos. BMC Genomics.

[25] Chernikova SB, Dorth JA, Razorenova OV, Game JC, Brown JM (2010). Deficiency in Bre1 impairs homologous recombination repair and cell cycle checkpoint response to radiation damage in mammalian cells. Radiat Res.

[26] Kari V, Shchebet A, Neumann H, Johnsen SA (2011). The H2B ubiquitin ligase RNF40 cooperates with SUPT16H to induce dynamic changes in chromatin structure during DNA double-strand break repair. Cell Cycle.

[27] Moyal L, Lerenthal Y, Gana-Weisz M, Mass G, So S, Wang SY (2011). Requirement of ATM-dependent monoubiquitylation of histone H2B for timely repair of DNA double-strand breaks. Mol Cell.

[28] Nakamura K, Kato A, Kobayashi J, Yanagihara H, Sakamoto S, Oliveira DV (2011). Regulation of homologous recombination by RNF20-dependent H2B ubiquitination. Mol Cell.

[29] Davie JR, Murphy LC (1990). Level of ubiquitinated histone H2B in chromatin is coupled to ongoing transcription. Biochemistry.

[30] Henry KW, Wyce A, Lo WS, Duggan LJ, Emre NC, Kao CF (2003). Transcriptional activation via sequential histone H2B ubiquitylation and deubiquitylation, mediated by SAGA-associated Ubp8. Genes Dev.

[31] Pavri R, Zhu B, Li G, Trojer P, Mandal S, Shilatifard A (2006). Histone H2B monoubiquitination functions cooperatively with FACT to regulate elongation by RNA polymerase II. Cell.

[32] Weinberger L, Voichek Y, Tirosh I, Hornung G, Amit I, Barkai N (2012). Expression noise and acetylation profiles distinguish HDAC functions. Mol Cell.

[33] Trujillo KM, Osley MA (2012). A role for H2B ubiquitylation in DNA replication. Mol Cell.

[34] Batta K, Zhang Z, Yen K, Goffman DB, Pugh BF (2011). Genome-wide function of H2B ubiquitylation in promoter and genic regions. Genes Dev.

[35] Pirngruber J, Shchebet A, Schreiber L, Shema E, Minsky N, Chapman RD (2009). CDK9 directs H2B monoubiquitination and controls replication-dependent histone mRNA 3'-end processing. EMBO Rep.

[36] Jung I, Kim SK, Kim M, Han YM, Kim YS, Kim D (2012). H2B monoubiquitylation is a 5'-enriched active transcription mark and correlates with exon-intron structure in human cells. Genome Res.

[37] Vitaliano-Prunier A, Babour A, Herissant L, Apponi L, Margaritis T, Holstege FC (2012). H2B ubiquitylation controls the formation of export-competent mRNP. Mol Cell.

[38] Herissant L, Moehle EA, Bertaccini D, Van Dorsselaer A, Schaeffer-Reiss C, Guthrie C (2014). H2B ubiquitylation modulates spliceosome assembly and function in budding yeast. Biol Cell.

[39] Long L, Thelen JP, Furgason M, Haj-Yahya M, Brik A, Cheng D (2014). The U4/U6 recycling factor SART3 has histone chaperone activity and associates with USP15 to regulate H2B deubiquitination. J Biol Chem.

[40] Xu Z, Song Z, Li G, Tu H, Liu W, Liu Y (2016). H2B ubiquitination regulates meiotic recombination by promoting chromatin relaxation. Nucleic Acids Res.

[41] Li X, Jiang Y, Ji Z, Liu Y, Zhang Q (2015). BRHIS1 suppresses rice innate immunity through binding to monoubiquitinated H2A and H2B variants. EMBO Rep.

[42] Chen S, Li J, Wang DL, Sun FL (2012). Histone H2B lysine 120 monoubiquitination is required for embryonic stem cell differentiation. Cell research.

[43] Chen S, Jing Y, Kang X, Yang L, Wang DL, Zhang W (2017). Histone H2B monoubiquitination is a critical epigenetic switch for the regulation of autophagy. Nucleic Acids Res.

[44] Kim J, Guermah M, McGinty RK, Lee JS, Tang Z, Milne TA (2009). RAD6-Mediated transcription-coupled H2B ubiquitylation directly stimulates H3K4 methylation in human cells. Cell.

[45] Fuchs G, Hollander D, Voichek Y, Ast G, Oren M (2014). Cotranscriptional histone H2B monoubiquitylation is tightly coupled with RNA polymerase II elongation rate. Genome Res.

[46] Cheng H, Guo Y, Yu Q, Zhou R (2003). The rice field eel as a model system for vertebrate sexual development. Cytogenet Genome Res.

[47] Harrison E, Trexler JC, Collins TM, Vazquez-Dominguez E, Razo-Mendivil U, Matamoros WA (2014). Genetic evidence for multiple sources of the non-native fish Cichlasoma urophthalmus (Gunther; Mayan Cichlids) in southern Florida. PLoS One.

[48] Zhao X, Luo M, Li Z, Zhong P, Cheng Y, Lai F (2018). Chromosome-scale assembly of the Monopterus genome. Gigascience.

[49] Lin S, Garcia BA (2012). Examining histone posttranslational modification patterns by high-resolution mass spectrometry. Methods Enzymol.

[50] Sheng Y, Song Y, Li Z, Wang Y, Lin H, Cheng H (2018). RAB37 interacts directly with ATG5 and promotes autophagosome formation via regulating ATG5-12-16 complex assembly. Cell Death Differ.

[51] Li Z, Li H, Xu X, Wang L, Liu B, Zheng W (2020). Haploinsufficiency of GCP4 induces autophagy and leads to photoreceptor degeneration due to defective spindle assembly in retina. Cell Death Differ.

[52] Ji H, Zhou Y, Zhuang X, Zhu Y, Wu Z, Lu Y (2019). HDAC3 Deficiency Promotes Liver Cancer through a Defect in H3K9ac/H3K9me3 Transition. Cancer Res.

[53] Raisner R, Kharbanda S, Jin L, Jeng E, Chan E, Merchant M (2018). Enhancer Activity Requires CBP/P300 Bromodomain-Dependent Histone H3K27 Acetylation. Cell reports.

[54] Osley MA (2006). Regulation of histone H2A and H2B ubiquitylation. Brief Funct Genomic Proteomic.

[55] Stillman B (2018). Histone Modifications: Insights into Their Influence on Gene Expression. Cell.

[56] Raymond CS, Murphy MW, O'Sullivan MG, Bardwell VJ, Zarkower D (2000). Dmrt1, a gene related to worm and fly sexual regulators, is required for mammalian testis differentiation. Genes Dev.

[57] Jin Q, Yu LR, Wang L, Zhang Z, Kasper LH, Lee JE (2011). Distinct roles of GCN5/PCAF-mediated H3K9ac and CBP/p300-mediated H3K18/27ac in nuclear receptor transactivation. Embo J.

[58] Wood K, Tellier M, Murphy S (2018). DOT1L and H3K79 Methylation in Transcription and Genomic Stability. Biomolecules.

[59] Yu Y, Liu L, Li X, Hu X, Song H (2019). The histone H4K20 methyltransferase PR-Set7 fine-tunes the transcriptional activation of Wingless signaling in Drosophila. J Genet Genomics.

[60] Kim K, Lee B, Kim J, Choi J, Kim JM, Xiong Y (2013). Linker Histone H1.2 cooperates with Cul4A and PAF1 to drive H4K31 ubiquitylation-mediated transactivation. Cell reports.

[61] Ngollo M, Lebert A, Daures M, Judes G, Rifai K, Dubois L (2017). Global analysis of H3K27me3 as an epigenetic marker in prostate cancer progression. BMC cancer.

[62] Tamburri S, Lavarone E, Fernández-Pérez D, Conway E, Zanotti M, Manganaro D (2020). Histone H2AK119 Mono-Ubiquitination Is Essential for Polycomb-Mediated Transcriptional Repression. Mol Cell.

[63] Masuyama H, Yamada M, Kamei Y, Fujiwara-Ishikawa T, Todo T, Nagahama Y (2012). Dmrt1 mutation causes a male-to-female sex reversal after the sex determination by Dmy in the medaka. Chromosome Res.

[64] Smith CA, Roeszler KN, Ohnesorg T, Cummins DM, Farlie PG, Doran TJ (2009). The avian Z-linked gene DMRT1 is required for male sex determination in the chicken. Nature.

[65] Lei N, Heckert LL (2002). Sp1 and Egr1 regulate transcription of the Dmrt1 gene in Sertoli cells. Biol Reprod.

[66] Minsky N, Shema E, Field Y, Schuster M, Segal E, Oren M (2008). Monoubiquitinated H2B is associated with the transcribed region of highly expressed genes in human cells. Nat Cell Biol.

[67] Shieh GS, Pan CH, Wu JH, Sun YJ, Wang CC, Hsiao WC (2011). H2B ubiquitylation is part of chromatin architecture that marks exon-intron structure in budding yeast. BMC Genomics.

[68] Fleming AB, Kao CF, Hillyer C, Pikaart M, Osley MA (2008). H2B ubiquitylation plays a role in nucleosome dynamics during transcription elongation. Mol Cell.

