# Supplementary material for: Identification of Histone Modifications Reveals a Role of H2b Monoubiquitination in Transcriptional Regulation of dmrt1 in Monopterus albus
Source: Int J Biol Sci. 2021 May 11;17(8):2009–20. doi: 10.7150/ijbs.59347 (PMC8193266; doi:10.7150/ijbs.59347)
Supplement: Supplementary file 1 — Supplementary figures and tables. [file ijbsv17p2009s1.pdf]

Supplementary Materials

Supplemental 3 figures and 4 tables

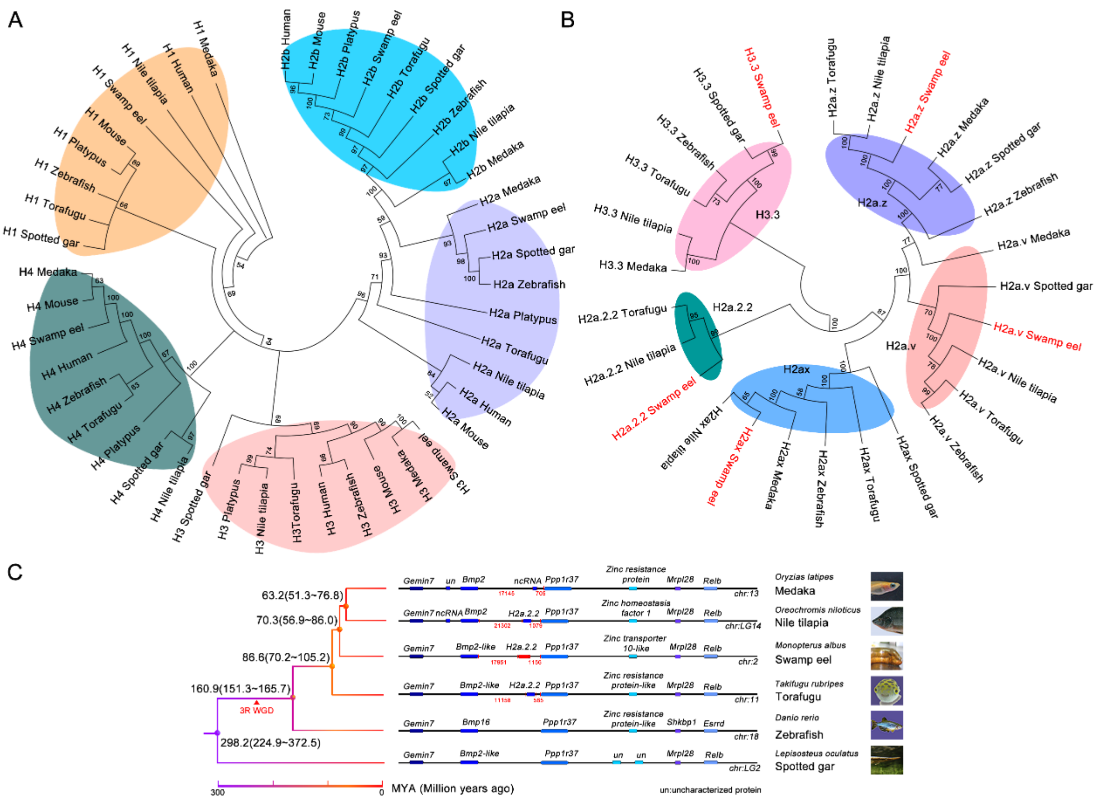

Figure S1: Phylogenetic tree of histones in vertebrates.

The phylogenetic tree was constructed using maximum-likelihood method based on the amino acid sequences of histones. (A) The histone in the phylogenetic tree were from human (*Homo sapiens*), mouse (*Mus musculus*), spotted gar (*Lepisosteus oculatus*), platypus (*Ornithorhynchus anatinus*), zebrafish (*Danio rerio*), tilapia (*Oreochromis niloticus*), medaka (*Oryzias latipes*), torafugu (*Takifugu rubripes*), and swamp eel (*Monopterus albus*). Five histone proteins of *Monopterus albus* were grouped into 5 clusters of the histone. (B) The histone variants in the phylogenetic tree were from the *Monopterus albus*, spotted gar (*Lepisosteus oculatus*), zebrafish (*Danio rerio*), torafugu (*Takifugu rubripes*), tilapia (*Oreochromis niloticus*), medaka (*Oryzias latipes*). Five histone variants proteins of *Monopterus albus* were grouped into 5 clusters of the histone variant. (C) Arrangement of *late histone h2a.2.2* on chromosome 2 of *Monopterus albus*, and the relative positions of these genes on the corresponding chromosomes in several related species. From top to bottom species are the medaka, tilapia, swamp eel, torafugu, zebrafish and spotted gar. The protein sequences above were from NCBI and Ensemble.

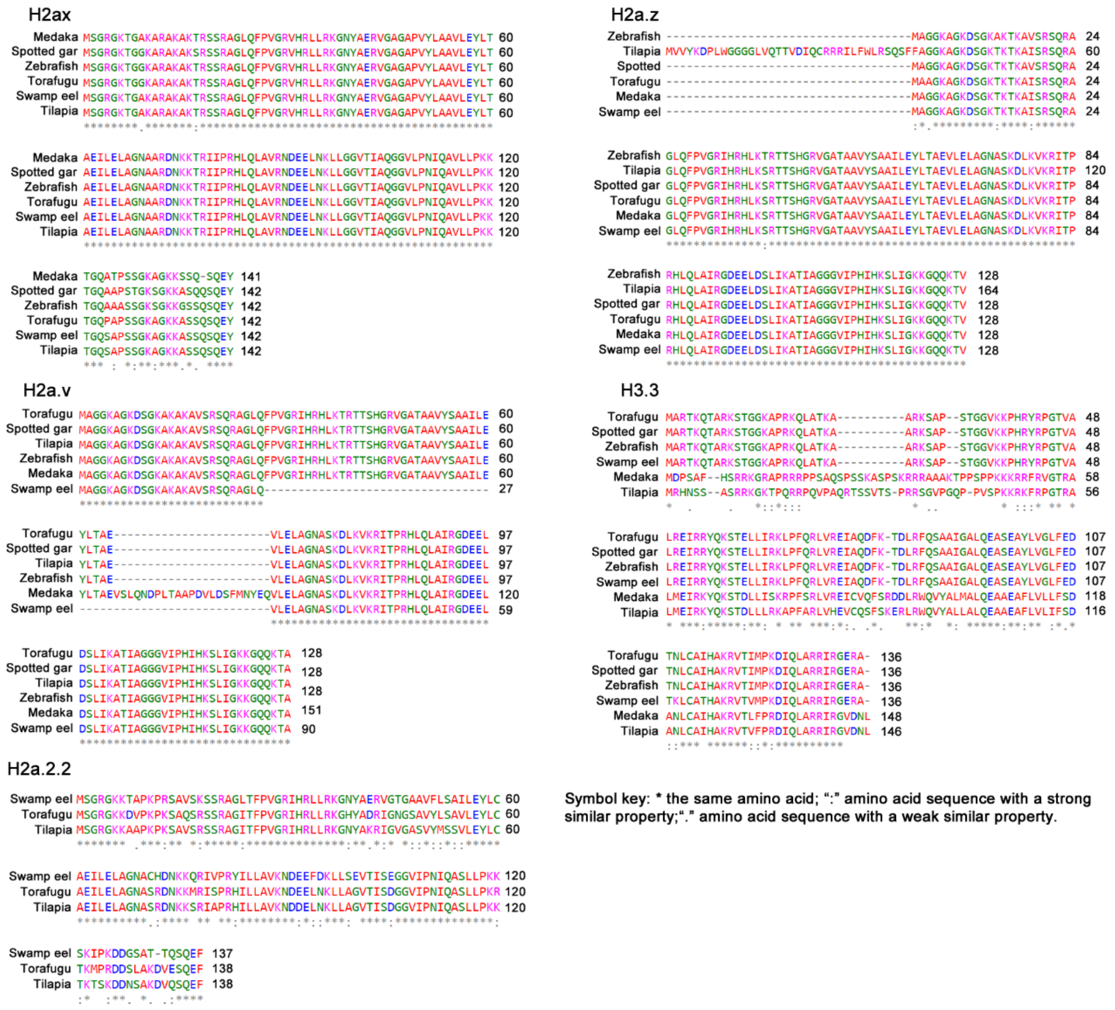

Figure S2: Homologous alignment of fish histone variants.

Using Clustal Omega (https://www.ebi.ac.uk/Tools/msa/clustalo/) to comparison of histone variants of Swamp eel (*Monopterus albus*) with its homologs from other fishes, such as, spotted gar (*Lepisosteus oculatus*), zebrafish (*Danio rerio*), torafugu (*Takifugu rubripes*), tilapia (*Oreochromis niloticus*), medaka (*Oryzias latipes*). Five histone variants proteins of *Monopterus albus* were grouped into 5 clusters of the histone variant. Symbol key: * for the same amino acid; ":" amino acid sequence with a strong similarity; "." Amino acid sequence with a weak similarity.

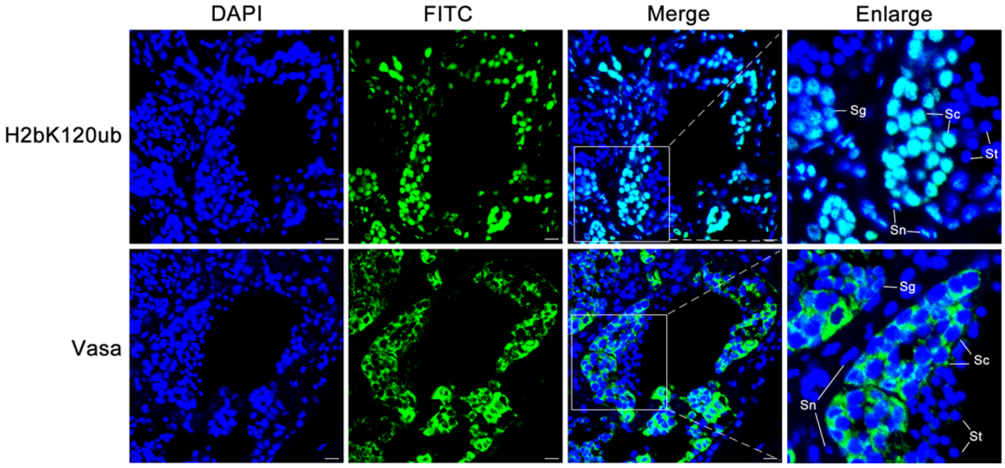

Figure S3: Expression of H2bK120ub in testis of *Monopterus albus*.

Immunofluorecent localization of H2bK120ub and Vasa proteins in testis using anti-H2bK120ub and anti-Vasa antibody. H2bK120ub was expressed in the nuclei of Sertoli cells and germ cells in testis, while Vasa was mainly expressed in the cytoplasm of germ cells. The nuclei were stained by DAPI. Sn, Sertoli cells; Sg, spermatogonia; Sc, spermatocytes; St, spermatids. Scale bar: 10 μm.

Table S1. The accession number of histones in the study

Gene Name	Species	Accession number
H2A	human	NM_003517
	mouse	NM_175658
	medaka	XM_004083246
	platypus	XM_001512321
	zebrafish	XM_002666942
	tilapia	XM_003459524
	spotted gar	XM_006642306
	torafugu	XM_003967840
	swamp eel	XM_020625504
H2B	human	NM_003528
	mouse	NM_175663
	medaka	XM_023954407
	platypus	XM_003430276
	zebrafish	XM_017357172
	tilapia	XM_025910855
	spotted gar	XM_006642351
	torafugu	XM_029843023
	swamp eel	XM_020620624
H3	human	NM_021059
	mouse	NM_013550
	medaka	XM_023955270
	platypus	XM_029055498
	zebrafish	NM_212996
	tilapia	XM_019354332
	spotted gar	XM_006638975
	torafugu	XM_003967832
	swamp eel	MT_188671
H4	human	NM_003548
	mouse	NM_178192
	medaka	XM_004065522
	platypus	XM_029055183
	zebrafish	XM_002662197
	tilapia	XM_025902644
	spotted gar	XM_015355476
	torafugu	XM_011621509
	swamp eel	XM_020587344
H1	human	NM_005322
	mouse	NM_008197
	medaka	XM_004072237
	platypus	XM_001516566

	zebrafish	NM_199552
	tilapia	XM_003450634
	spotted gar	XM_015359971
	torafugu	XM_003961302
	swamp eel	XM_020613492

Table S2. The accession number of histone variants in the study

Gene Name	Species	Accession number
H2ax	tilapia	XM_003446866
	zebrafish	NM_201073
	torafugu	XM_003971139
	medaka	XM_004076333
	spotted gar	XM_015337319
	swamp eel	XM_020598961
H2a.v	tilapia	XM_019365876
	zebrafish	NM_153644
	torafugu	XM_003975077
	medaka	XM_024274880
	spotted gar	XM_015340344
	swamp eel	XM_020585777
H2a.z	tilapia	XM_005464173
	zebrafish	NM_001043323
	torafugu	XM_003966753
	medaka	XM_004080073
	spotted gar	XM_015345056
	swamp eel	XM_020594837
H2a.2.2	tilapia	XM_019355513
	zebrafish	--
	torafugu	XM_003968358
	medaka	--
	spotted gar	--
	swamp eel	XM_020624512
H3.3	tilapia	XM_003439500
	zebrafish	NM_001017599
	torafugu	XM_029848527
	medaka	XM_023960501
	spotted gar	XM_015340704
	swamp eel	XM_020625364

Table S3. The primers in the study.

Genes/fragments	Primer sequence (5' - 3')	Tm (℃)
H2a-F	ATGTCTGGTAGAGGGAAAACCG	58
H2a-R	TCACTTGCTCTTTGCTGGTTTC	
H2b-F	ATGCCTGATCCTGCGAAGTC	56
H2b-R	CTATTTTGAGCTGGTGTACTTGGTC	
H3-F	ATGGCAAGAACCAAGCAGAC	55
H3-R	CTATGCCCTTTCTCCGCG	
H4-F	ATGAGCGGAAGAGGAAAAGG	57
H4-R	TTAGCCTCCGAAGCCGTACA	
dmrt1-F	GCCAGTGCTCCAAATGTAA	58
dmrt1-R	GCAGCAGGAGAAGAAGTCG	
hprt-F	ATGGCGACATCCAGCTCCTG	60
hprt-R	TCTGGCCGGTAGCCAACACT	

Table S4. The histone modifications sites in the study.

H2a modified sites. "+", detected; "-", not detected.

Residue/Peptide	Modification	Ovary	Ovotestis	Testis
SSR$_{20}$AGLQFPVGR	Unmodified	+	+	+
	R20 Methylation	-	+	+
LLRK$_{36}$GNYAER	Unmodified	+	+	+
	K36 Methylation	-	-	+
	K36 Di-methylation	-	-	+
HLQLAVR$_{88}$NDEELNK$_{95}$	Unmodified	+	+	+
	R88 Di-methylation	+	+	+
	K95 Acetylation	-	-	+
	K95 Methylation	-	-	+
	K95 Di-methylation	-	-	+
	K95 Tri-methylation	-	-	+
LLGGVTIAQGGVLPNIQAV LLPK$_{118}$K$_{119}$TEK$_{122}$PAK$_{125}$	Unmodified	+	+	+
	K118 Methylation	-	-	+
	K118 Di-methylation	-	-	+

	K118 Ubiquitination	-	-	+
	K119 Acetylation	-	-	+
	K119 Methylation	-	-	+
	K119 Ubiquitination	-	-	+
	K122 Di-methylation	-	-	+
	K125 Di-methylation	-	-	+

H2b modified sites. "+", detected; "-", not detected.

Residue/Peptide	Modification	Ovary	Ovotestis	Testis
K$_{31}$TR$_{33}$K$_{34}$ESYAIYVYK	Unmodified	+	+	+
	K31 Methylation	-	+	-
	R33 Methylation	+	+	+
	K34 Acetylation	-	+	+
	K34 Methylation	+	+	+
	K34 Di-methylation	+	+	+
	K34 Ubiquitination	-	-	+
KESYAIYVYK$_{43}$VLK$_{46}$	Unmodified	+	+	+
	K43 Acetylation	-	-	+
	K43 Methylation	-	-	+
	K43 Di-methylation	-	-	+
	K43 Ubiquitination	+	+	+
	K46 Acetylation	+	+	+
	K46 Methylation	+	+	+
	K46 Di-methylation	-	+	+
	K46 Ubiquitination	+	+	+
VLKQVHPDTGISSK$_{57}$	Unmodified	+	+	+
	K57 Acetylation	-	-	+
	K57 Methylation	-	-	+
	K57 Di-methylation	-	-	+
	K57 Ubiquitination	-	-	+
IAGEASR$_{79}$LAHYNK$_{85}$	Unmodified	+	+	+
	R79 Methylation	-	-	+
	K85 Ubiquitination	-	+	+
STITISR$_{92}$EIQTAVR$_{99}$	Unmodified	+	+	+
	R92 Methylation	-	-	+
	R99 Methylation	-	-	+
LLLPGELAK$_{108}$HAVSEGTK$_{116}$	Unmodified	+	+	+
	K108 Acetylation	+	+	+
	K108 Di-methylation	-	-	+
	K108 Tri-methylation	-	-	+
	K108 Ubiquitination	+	+	+
	K116 Acetylation	+	+	-
	K116 Di-methylation	-	-	+
	K116 Ubiquitination	-	+	+

HAVSEGTKAVTK$_{120}$YTSSK	Unmodified	+	+	+
	K120 Ubiquitination	-	+	+

H3 modified sites. "+", detected; "-", not detected.

Residue/Peptide	Modification	Ovary	Ovotestis	Testis
K$_9$STGGK$_{14}$APR	Unmodified	+	+	+
	K9 Acetylation	-	-	+
	K14 Acetylation	-	-	+
K$_{18}$QLATK$_{23}$AAR	Unmodified	+	+	+
	K18 Acetylation	+	+	+
	K18 Di-methylation	-	+	-
	K23 Acetylation	+	+	+
K$_{27}$SAPATGGVK$_{36}$K$_{37}$	Unmodified	+	+	+
	K27 Acetylation	-	+	+
	K27 Methylation	-	-	+
	K27 Di-methylation	-	+	+
	K27 Tri-methylation	-	-	+
	K36 Acetylation	-	+	+
	K36 Methylation	-	+	+
	K36 Di-methylation	-	+	+
	K37 Acetylation	-	+	+
	K37 Methylation	-	+	+
	K37 Di-methylation	-	+	+
R$_{53}$YQK$_{56}$STELLIR	Unmodified	+	+	+
	R53 Acetylation	-	+	+
	R53 Methylation	-	+	+
	K56 Acetylation	-	+	+
	K56 Methylation	-	+	+
	K56 Di-methylation	-	+	-
	K56 Ubiquitination	+	+	+
LVREIAQDFK$_{79}$	Unmodified	+	+	+
	K79 Acetylation	+	+	+
	K79 Methylation	+	+	+
	K79 Di-methylation	+	+	+
	K79 Ubiquitination	-	+	+
VTIMPK$_{122}$DIQLAR$_{128}$	Unmodified	+	+	+
	K122 Acetylation	-	+	+
	K122 Ubiquitination	+	+	+
	K128 Methylation	-	-	+

H4 modified sites. "+", detected; "-", not detected.

Residue/Peptide	Modification	Ovary	Ovotestis	Testis
GK$_5$GGK$_8$GLGK$_{12}$GGAK$_{16}$	Unmodified	+	+	+
	K5 Acetylation	+	+	+

	K8 Acetylation	+	+	+
	K12 Acetylation	+	+	+
	K16 Acetylation	+	+	+
$K_{20}VLR_{23}DNIQGITK_{31}PAIR$	Unmodified	+	+	+
	K20 Acetylation	+	+	+
	K20 Methylation	-	-	+
	K20 Di-methylation	+	+	+
	K20 Ubiquitination	+	-	-
	R23 Acetylation	+	+	+
	R23 Methylation	-	-	+
	R23 Di-methylation	+	+	+
	R23 Tri-methylation	+	+	+
	K31 Di-methylation	+	-	-
	K31 Ubiquitination	+	-	+
$RISGLIYEETR_{55}GVLK_{59}$	Unmodified	+	+	+
	R55 Di-methylation	+	+	+
	K59 Acetylation	-	-	+
	K59 Di-methylation	-	+	-
	K59 Ubiquitination	+	+	+
$VFLENVIR_{67}$	Unmodified	+	+	+
	K67 Methylation	-	+	-
	K67 Di-methylation	+	+	+
$DAVTYTEHAK_{77}$	Unmodified	+	+	+
	K77 Acetylation	+	+	+
	K77 Di-methylation	+	+	+
	K77 Ubiquitination	+	+	+
$R_{78}K_{79}TVTAMDVVYALK_{91}$	Unmodified	+	+	+
	R78 Acetylation	-	+	+
	R78 Methylation	+	+	+
	K79 Acetylation	+	+	+
	K79 Methylation	+	-	+
	K79 Di-methylation	-	+	+
	K79 Ubiquitination	+	+	+
	K91 Acetylation	-	-	+
	K91 Di-methylation	+	-	-
	K91 Ubiquitination	+	+	+
